# MS4A1-PTGS2 axis induces taurine metabolic reprogramming to exacerbate abdominal aortic aneurysm progression

**DOI:** 10.7150/ijms.99659

**Published:** 2024-08-06

**Authors:** Xuejun Sun, Chaoxiang Du, Ye Chen, Zhibin Cai, Liangwan Chen

**Affiliations:** 1 Department of Cardiovascular Surgery, Fujian Medical University Union Hospital, Fuzhou City, Fujian Province, 350001, China.; 2 Department of Cardiovascular Surgery, Quanzhou First Hospital Affiliated to Fujian Medical University, Quanzhou, 362002, China.; 3 Zhongshan Hospital (Xiamen), Fudan University, Xiamen, 361015, China.; 4 Chinese PLA General Hospital, Beijing, China.; 5 Fujian Provincial Clinical Research Center for Cardiovascular Diseases, Heart Center of Fujian Medical University, Fuzhou City, Fujian Province, 350001, China.; 6 Fujian Provincial Center for Cardiovascular Medicine, Fuzhou City, Fujian Province, 350001, China.

**Keywords:** Taurine Metabolic Reprogramming, Abdominal Aortic Aneurysm, Single-cell RNA sequencing, Weighted Gene Co-expression Network Analysis, Metabolomic Analysis

## Abstract

This study unveils the pivotal roles of taurine metabolic reprogramming and its implications in the development and progression of Abdominal Aortic Aneurysm (AAA). Leveraging an integrated approach that combines single-cell RNA sequencing (scRNA-seq) and Weighted Gene Co-expression Network Analysis (WGCNA), our research investigates the intricate transcriptional and gene expression dynamics crucial to AAA. Our findings uniquely link metabolic shifts to the integrity of the extracellular matrix (ECM) and the functionality of smooth muscle cells (SMCs), key elements in the pathology of AAA. Utilizing scRNA-seq data from a mouse model (GSE152583 dataset), we identified critical alterations in cellular composition during AAA progression, particularly highlighting shifts in fibroblasts and inflammatory cells. Concurrently, WGCNA of human AAA tissue samples has outlined distinct gene expression patterns correlated with disease severity and progression, offering comprehensive insights into both molecular and cellular disease mechanisms. Moreover, this study introduces innovative metabolic profiling techniques to identify differential metabolites in AAA, integrating extensive metabolomic analyses with pathway enrichment strategies. This novel approach has pinpointed potential biomarkers and therapeutic targets, notably within taurine metabolism pathways, crucial for crafting non-surgical interventions. By merging state-of-the-art bioinformatics with thorough molecular analysis, our study not only enhances the understanding of AAA's complex pathophysiology but also catalyzes the development of targeted therapeutic strategies. This research represents a significant advancement in the molecular characterization of AAA, with substantial implications for its future diagnosis and treatment strategies.

## Introduction

Abdominal aortic aneurysm (AAA) is a peripheral vascular disease characterized by localized dilation of the abdominal aorta 1. Often occurring without warning, AAA ruptures result in a mortality rate of up to 90%, making it one of the deadliest vascular diseases. Epidemiological studies indicate that the positive detection rate of AAA among the population over 60 years old is close to 1%, with the incidence rising annually as the population ages 2, 3. Currently, surgical intervention is the sole method for managing AAA. Due to its invasive nature and high risk, surgery is typically reserved for cases where the aneurysm diameter exceeds 50mm and the risk of rupture significantly increases. However, most patients are diagnosed in the early stages of AAA when the aneurysm is too small to meet surgical criteria 1. There is a stark absence of effective treatments to curb or slow the progression of these smaller AAAs, posing a severe threat to patient health and well-being 4, 5.

AAA is considered a degenerative vascular disease, yet its specific etiology and pathogenesis remain unclear. Historically, the degradation of the extracellular matrix (ECM) and the apoptosis of smooth muscle cells (SMCs) have been recognized as the hallmark pathological features of AAA. Recent research over the past decade has revealed that chronic inflammation plays a pivotal role in the progression of AAA 4, 5. It contributes to the destruction of the aortic media and the apoptosis and dysfunction of SMCs through the release of a series of proteolytic enzymes and cytokines. This process drives expansive remodeling of the aortic wall, progressive functional decline, and the formation of intraluminal thrombi 2. Against this backdrop, this study will specifically examine the role of metabolic reprogramming in the development and progression of AAA. Metabolic reprogramming refers to the process by which cells alter their metabolic pathways to adapt to environmental stresses, such as hypoxia or inflammation, and is a known critical pathological process in various diseases, including cancer and cardiovascular diseases 6, 7. In AAA, metabolic reprogramming may indirectly affect the stability of the ECM and the viability of SMCs by modulating energy metabolism, oxidative stress responses, and cellular survival pathways 5.

By thoroughly investigating the link between metabolic reprogramming and AAA, this research aims to unveil new pathological mechanisms of AAA and explore potential early intervention strategies 2, 4. This understanding is not only expected to deepen our comprehension of the complex pathophysiological processes of AAA but also to provide a theoretical basis and experimental evidence for developing non-surgical treatment options. The findings of this study could offer new perspectives and strategies for the prevention and treatment of AAA, particularly in controlling the early progression of the disease and extending patient survival.

## Methods

### Ethical statement

This study adhered to the Declaration of Helsinki and the ethical guidelines of Fujian Medical University Union Hospital. The Ethics Committee approved the experimental protocols, with the reference number GSE152583.

### scRNA analysis of abdominal aortic aneurysm mouse model

In this study, we employed scRNA-seq techniques to thoroughly analyze the transcriptomic profiles of single cells in a mouse model of abdominal aortic aneurysm. Initially, we downloaded the GSE152583 dataset from the Gene Expression Omnibus (GEO; https://www.ncbi.nlm.nih.gov/geo/query/acc.cgi) [8]and conducted data preprocessing using Seurat, which included filtering cells based on gene expression and mitochondrial content (minimum of 500 genes, maximum of 5000 genes, and mitochondrial gene percentage capped at 15%) 9. To reduce batch effects and variability in expression levels, we integrated and normalized the data using the Harmony algorithm 10. Additionally, we applied the limma package to analyze differentially expressed genes across various conditions and time points 11. For precise cell type annotation, we utilized the SingleR and celldex packages 10, classifying cell populations based on known markers. Through these methodologies, we significantly enhanced the interpretability of our data and laid the foundation for further sub-cluster analysis, revealing critical cell subpopulations and transcriptional changes during the progression of the aneurysm.

### Weighted Gene Co-expression Network Analysis (WGCNA) analysis

In this segment of our study, we conducted a WGCNA 12 on data sourced from GSE232911^13^, which comprises genome-wide expression profiles from 246 samples of media and adventitia, both thrombus-covered and thrombus-free, from the abdominal aortic vessel walls of 76 patients with AAA and 13 organ donor controls. We utilized original CEL chip data, corresponding clinical characteristics of the samples, and annotation files specific to the Affymetrix Human Transcriptome Array 2.0 platform 11. The data preprocessing included utilizing the 'affy' and 'limma' packages for robust multi-array averaging (RMA) background correction, normalization, and gene annotation 11. This was followed by differential expression analysis to identify significant transcriptomic variations. For the WGCNA, we focused on the top 25% most variable genes, correlating their expression profiles with clinical features to construct a network 12. We analyzed the core modules derived from this network, facilitating an understanding of the key gene interactions and pathways potentially involved in AAA. This approach enabled the identification of pivotal biomolecular pathways that may underlie the pathological variations observed in AAA, further advancing our understanding of the disease's molecular mechanisms.

### Construction of protein-protein interaction network and pathway interaction network

In the subsequent phase of our analysis, protein-protein interaction (PPI) networks were explored utilizing the STRING database (Version 12.0; available at https://string-db.org) 14. This analysis facilitated the mapping of intricate protein interactions which are crucial for understanding the molecular underpinnings of abdominal aortic aneurysm. Following the construction of the PPI network, Cytoscape software (Version 3.10.2; available at https://cytoscape.org) 15, 16 was employed to analyze the network's degree of connectivity, allowing for the identification of core nodes within the network. These core nodes represent significant proteins that play key roles in the biological processes associated with the disease. Finally, pathway interaction analyses were performed using the Metascape online tool (available at https://metascape.org/gp/index.html) 17, employing a minimum overlap of 3, a p-value cutoff of 0.05, and a minimum enrichment of 1.5. This step was essential for identifying significant biological pathways and interactions enriched among the proteins in the core network modules, providing deeper insights into the potential therapeutic targets for AAA.

### Full spectrum metabolome detection and analysis

In this study, we incorporated metabolomic analysis using a label-free, untargeted approach on 10 AAA specimens and 7 normal abdominal aorta specimens. All protocols were approved by the Ethics Committee of Fujian Medical University Union Hospital (No. 2022KY031). Metabolomic expression level analysis was primarily conducted on the abundance of metabolites, with statistical evaluation of metabolite expression to assess intra-group and inter-group correlations of metabolite expression characteristics 18. Initially, raw data were processed using library search analysis through the Compound Discoverer software, a commercial metabolomic library analysis tool developed by Thermo Scientific. Compound Discoverer 3.0 simplifies the identification of unknown compounds, measures real differences between samples, and elucidates biological pathways 19. This software conducts spectral library searches with the mzCloud and internal Thermo Scientific mzVault libraries, enabling faster identification of unknown analytes 20. It calculates statistical differences across multiple sample groups, identifies impurities, metabolites, and degradation products in complex samples, and performs fluxomics experiments, pathway visualization, and mapping of detected compounds and flux information onto pathways 18, 20. Principal Component Analysis (PCA) is a widely used dimension reduction technique. By performing orthogonal transformations using quantitative information of metabolites as variables, PCA transforms a large set of metabolite data into principal components, allowing for visual representation of sample differences through spatial data distribution 21. Partial Least Squares Discriminant Analysis (PLS-DA) and Orthogonal PLS-DA (OPLS-DA) are supervised discriminant statistical methods that establish a relationship model between metabolite expression and sample categories using PLS-DA, facilitating the prediction of sample categories. Models for pairwise group comparisons using PLS-DA or OPLS-DA are constructed, with model parameters provided in tabular format 21, 22.

### Identification of differential metabolites and pathway enrichment analysis

Differentially expressed metabolites refer to those whose expression levels are upregulated or downregulated between samples or within the same sample under different conditions. Typically, these are evaluated based on fold changes and statistical significance. In our analysis, metabolites with an absolute log fold change (logFC) greater than 1 and a p-value less than 0.05 were classified as differentially expressed. Furthermore, the names of these differentially expressed metabolites were submitted to the MetaboAnalyst database (https://www.metaboanalyst.ca/) 21, 23, 24 for functional enrichment and annotation, yielding enriched Kyoto Encyclopedia of Genes and Genomes (KEGG) pathways 25. We then filtered the enriched KEGG data, using a p-value cutoff of less than 0.05 as the standard for significance 26.

### Machine learning identification of core regulatory genes and pathways

The classification analysis of data samples based on differential gene expression in our study was conducted using a comprehensive suite of machine learning models, implemented in R version 4.3.2 and Python version 3.12. The models utilized include XGB Classifier, Logistic Regression, RandomForest Classifier, AdaBoost Classifier, DecisionTree Classifier, GradientBoosting Classifier, Gaussian NB, SVC, and KNeighbors Classifier. Each model was meticulously configured with specific parameters to enhance predictive accuracy and model robustness. For instance, the XGB Classifier was tuned with a learning rate of 0.3 and a max depth of 4, while the Logistic Regression used a regularization strength of 1.0 and a tolerance of 0.0001 for convergence. Similarly, RandomForest Classifier was optimized with 100 trees and a gini criterion for split quality assessment 27. The selection and tuning of these parameters were crucial for maximizing the discriminatory power of the models, ensuring the reliable classification of samples and providing insightful correlations between gene expression patterns and phenotypic traits. This approach underscores the integration of advanced computational techniques in genomic research, facilitating a deeper understanding of genetic influences on disease mechanisms 28.

### Partial correlation network analysis

Here, partial correlation network analysis was conducted on core gene and pathway expression levels using the 'sand' package in R 29. The direct associations between nodes were adjusted using the Benjamini-Hochberg correction method to control for false discovery rates. Additionally, molecular interactions of core regulatory genes were analyzed using the online tool available at https://zs-revelen.com 30. This approach facilitated a deeper understanding of the intricate relationships and regulatory mechanisms among the core genes within our dataset 31.

## Results

### scRNA analysis indicates that the absence of fibroblasts is a predominant cellular group in the onset and progression of AAA

**Figure 1:**
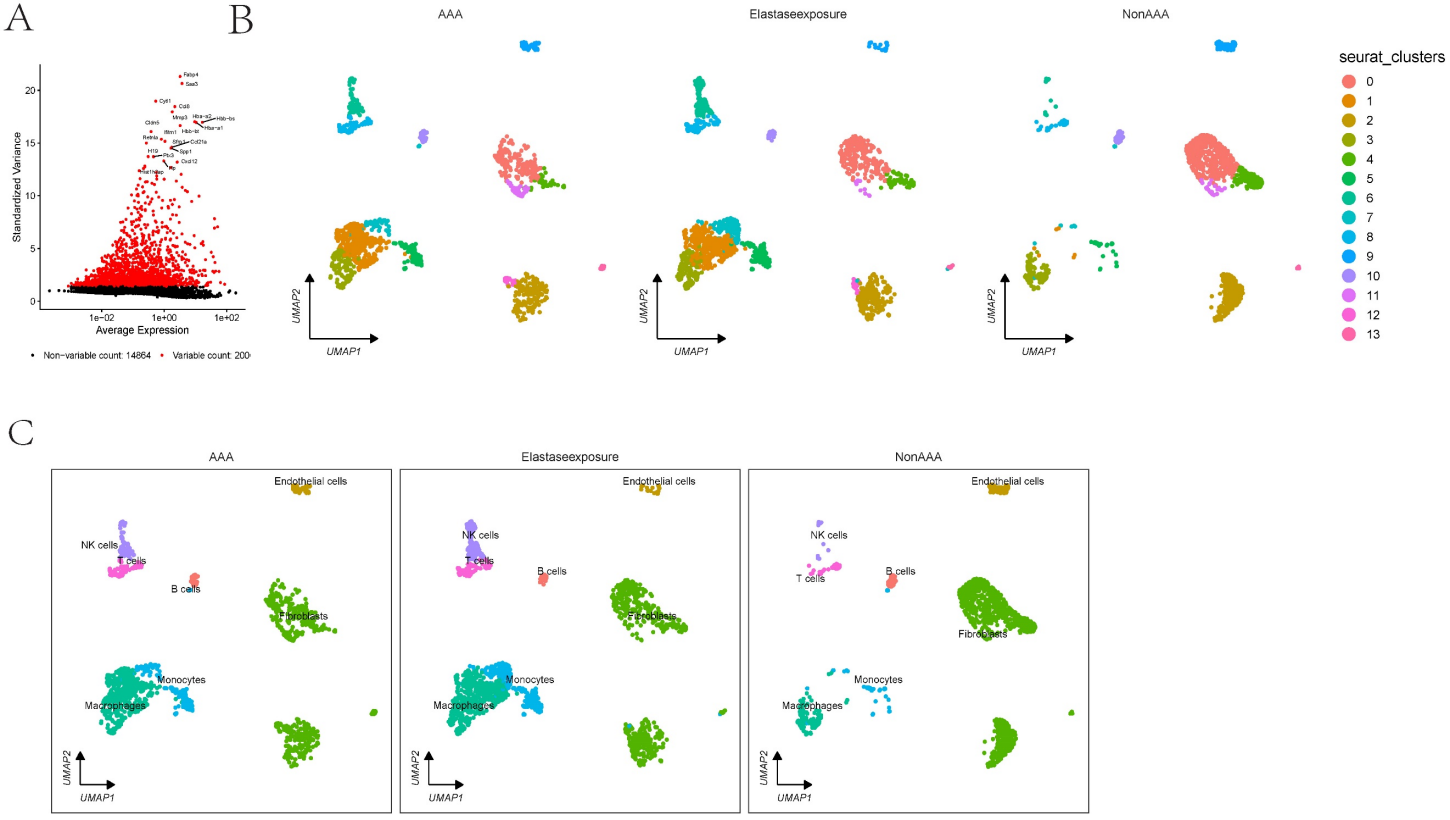
** The scRNA analysis of AAA dataset.** 1A showing the differential expression of selected markers and UMAP cluster analysis displaying cell type distribution in response to AAA Elastase exposure. Markers are plotted against their expression and variance, highlighting variable and non-variable counts. 1B-C showing the clusters in UMAP plots are annotated with cell types showing differences between conditions.

**Figure 2:**
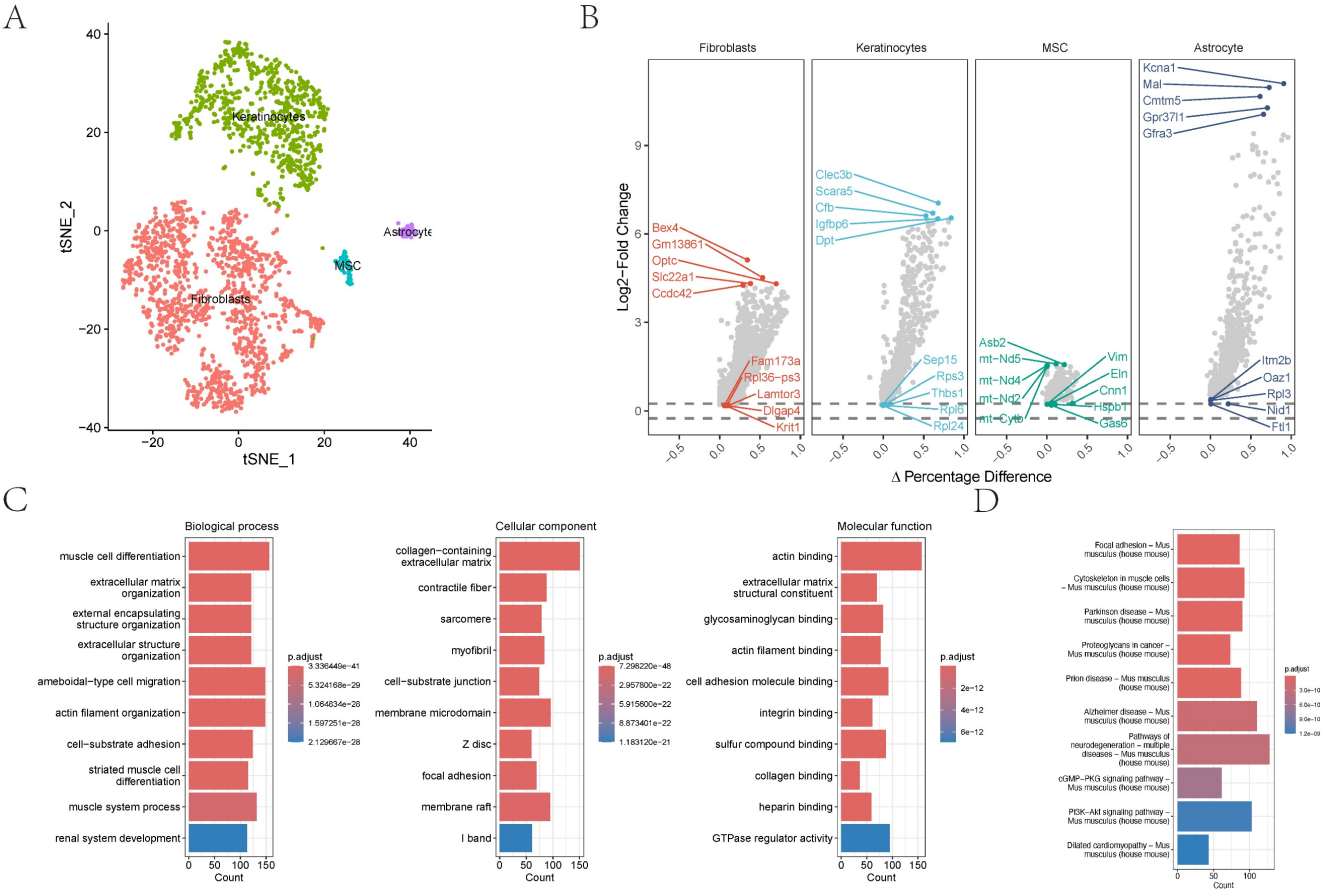
** The distribution and analysis of key cell types like fibroblasts and keratinocytes.** 2A-B showing the tSNE plot demonstrating cell type categorization and differential gene expression analysis. 2C showing the gene names and their expression differences are displayed alongside biological process alterations, quantified by gene counts and statistical significance.

In the GSE152583 dataset, the principal variant genes in AAA were identified as Fabp4, Saa3, Cytl1, Ccl8, and Mmp3, which are associated with fibrosis and inflammatory responses (Figure 1A). Cell subpopulations were categorized into fibroblasts, monocytes, macrophages, NK cells, B cells, T cells, and endothelial cells, among which fibroblasts, macrophages, monocytes, and endothelial cells were the core cell groups (Figure 1B-C). Further subclassification of fibroblasts revealed categories such as MSC, astrocyte, keratinocytes, and fibroblasts (Figure 2A). The primary upregulated genes in fibroblasts were Bex4, Gm13861, Optc, Slc22a1, and Ccdc42, while downregulated genes included Fam173a, Rpl36-ps3, Lamtor3, Dlgap4, and Krit1. Keratinocytes primarily upregulated genes such as Clec3b, Scara5, Cfb, Igfbp6, and Dpt, with downregulated genes including Sep15, Rps3, Thbs1, Rpl6, and Rpl24. Differential analysis yielded 1303 differential genes, with 1180 upregulated and 1923 downregulated genes (Figure 2B). GO analysis revealed enrichment in biological process terms like muscle cell differentiation (Adjusted p-value=3.37E-41), extracellular matrix organization (Adjusted p-value=1.24E-37), external encapsulating structure organization (Adjusted p-value=1.24E-37), extracellular structure organization (Adjusted p-value=1.33E-37), and ameboidal-type cell migration (Adjusted p-value=2.75E-36). Significantly enriched cellular component maps include collagen-containing extracellular matrix (Adjusted p-value=7.30E-48), contractile fiber (Adjusted p-value=7.49E-26), sarcomere (Adjusted p-value=1.42E-25), myofibril (Adjusted p-value=3.08E-25), and cell-substrate junction (Adjusted p-value=1.70E-24). Molecular function pathways notably enriched were actin binding (Adjusted p-value=1.42E-25), extracellular matrix structural constituent (Adjusted p-value=1.42E-25), glycosaminoglycan binding (Adjusted p-value=1.42E-25), actin filament binding (Adjusted p-value=1.42E-25), and cell adhesion molecule binding (Adjusted p-value=1.42E-25) (Figure 2C). Pathways such as focal adhesion (Adjusted p-value=1.87E-19), cytoskeleton in muscle cells (Adjusted p-value=1.87E-19), cGMP-PKG signaling pathway (Adjusted p-value=5.86E-10), and PI3K-Akt signaling were also significantly enriched.

### WGCNA analysis identifies core gene sets associated with vascular dilation and thrombosis

**Figure 3:**
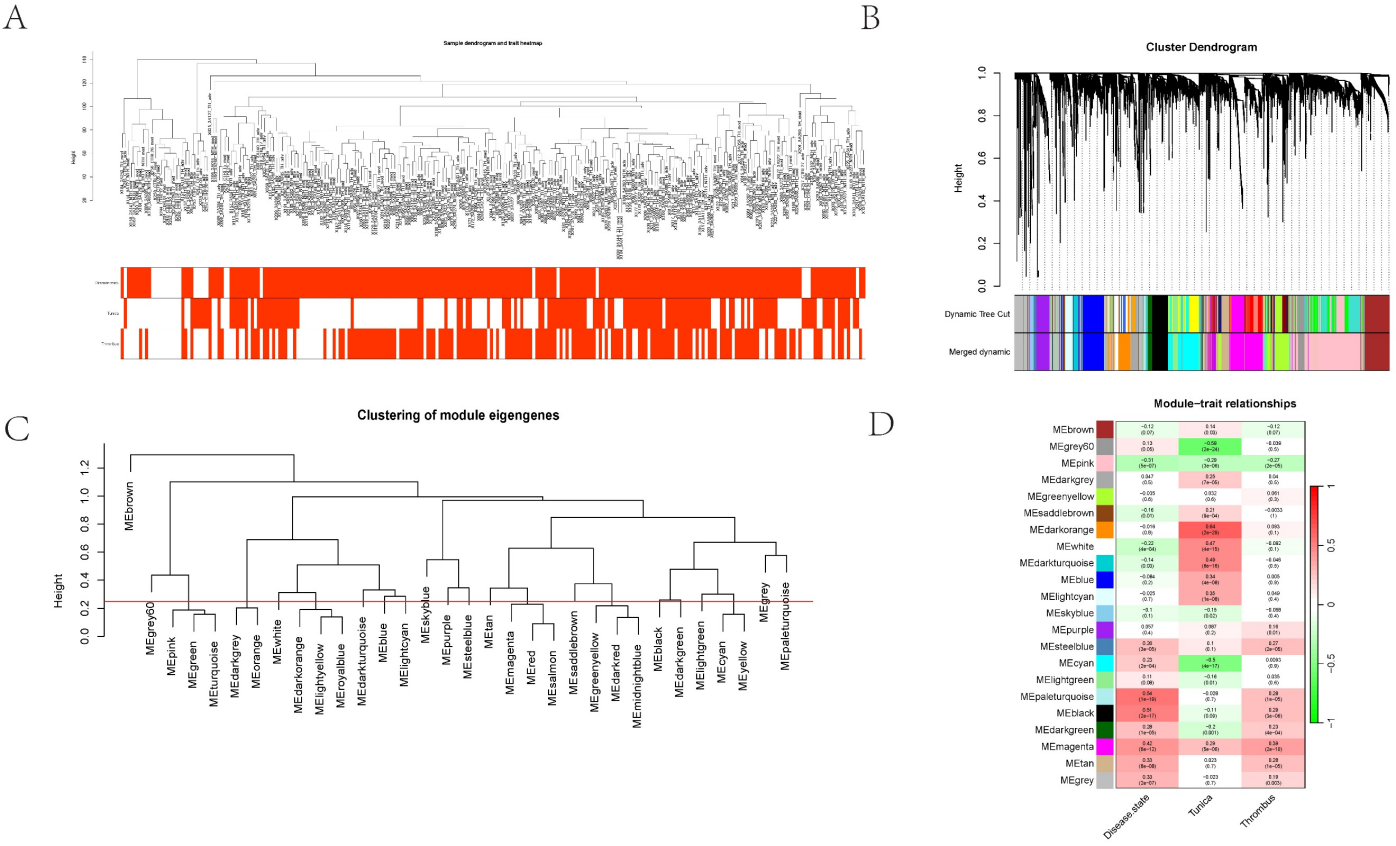
** The cluster dendrogram using hierarchical clustering methods to group different gene expression modules.** 3A showing the hierarchical clustering of gene modules and their relationships with various traits. 3B showing the cluster dendrogram using dynamic tree cut methods. 3C-D illustrated the dendrogram categorizes modules by similarity, and the heatmap details their correlations with traits such as disease state and other cellular components, complete with statistical annotations.

Initially, clustering was performed on the entire sample set, demonstrating associations with clinical features, notably with vascular tissue types (including adventitia or intima) and thrombosis (Figure 3A). Utilizing the dynamic tree cut algorithm, we identified 22 gene modules (Figure 3B). Cluster analysis of these modules revealed that MEbrow, MEskyblue, MEpaleturquoise, and MEgrey formed one cluster; other modules coalesced into another cluster (Figure 3C). Gene module-to-clinical feature association analysis showed that MEgrey60 (correlation coefficient = -0.59, p-value = 2e-24) and MEdarkorange (correlation coefficient = 0.64, p-value = 2e-29) were closely associated with vascular tissue types, MEpaleturquoise was closely linked to disease status (correlation coefficient = 0.54, p-value = 1e-19), and MEmagenta was closely related to aneurysm thrombosis (correlation coefficient = 0.39, p-value = 2e-10) (Figure 3D). Further analyses constructing core module protein-protein interaction and regulatory pathway networks revealed that the MEmagenta module genes are predominantly related to immune regulation, MEPaleturquoise module genes to metabolic anomalies, MEGrey60 module genes to extracellular matrix regulation, and MEDarkorange module genes to vasculature development.

### Full spectrum metabolomics mass spectrometry analysis

**Figure 5:**
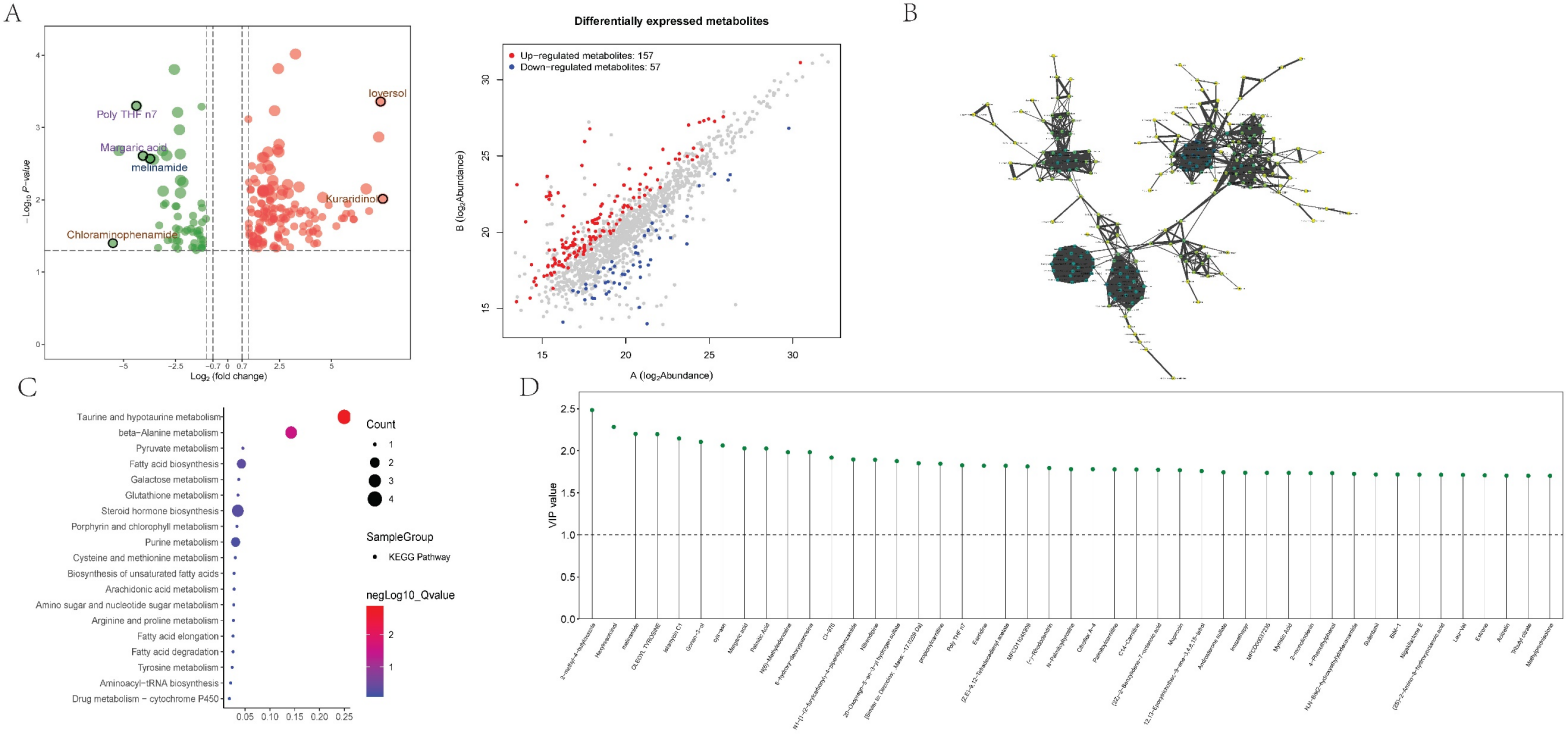
** The detection and enrichment analysis of differentially expressed metabolites.** 5A showing the analysis of metabolomic data showcasing differentially expressed metabolites. The plot differentiates between up-regulated and down-regulated metabolites based on log2 fold change and statistical significance, providing a comprehensive view of metabolic alterations. 5B represents the interaction network analysis of differential metabolites, 5C shows the results of pathway enrichment analysis for differential metabolites; 5D illustrates the distribution of core differential metabolites.

Subsequently, we conducted further differential and correlation analyses on metabolites in abdominal aortic vascular tissues from 17 human subjects. In the differential analysis, we identified 157 upregulated and 57 downregulated metabolites, and visualized them using a clustering heatmap (Figure 5A). In the correlation analysis, we employed a quantitative PLSDA scoring method to perform Pearson correlation analysis on the identified differential metabolites. The results indicated strong correlations between Hexylresorcinol and Palmitic Acid, Margaric Acid; between Bilirubin and NP-003145 as well as C18-Carnitine; and between Palmitic Acid and Margaric Acid. To further clarify the interactions among differential metabolites, we conducted an interaction analysis. The results identified 11 metabolites as hubs within the differential metabolites: Marimastat, Ozagrel, (1R,2S)-Tranylcypromine, 4-Methoxy DMT, Meclizine, Spermine, PEG n6, Melonal, Furaneol, 4-tert-Amylphenol, and Mexacarbate (Figure 5B). Pathway enrichment analysis indicated significant enrichment in core pathways such as Taurine and hypotaurine metabolism (Hits = 4; p-value = 0.001), beta-Alanine metabolism (Hits = 3; p-value = 0.028), and Pyruvate metabolism (Hits = 2; p-value = 0.045) (Figure 5C). Differential metabolites were typically evaluated and selected based on fold changes and significance levels, with criteria set as an absolute logFC >1 and p-value <0.05. Core differential metabolites identified included N1-[1-(2-furylcarbonyl)-4-piperidyl]benzamide, N(1)-acetylspermidine, OLEOYL TYROSINE, Ioversol, and Bilirubin (Figure 5D).

### Core pathway analysis of adventitia and intima in abdominal aortic vessels

**Figure 6:**
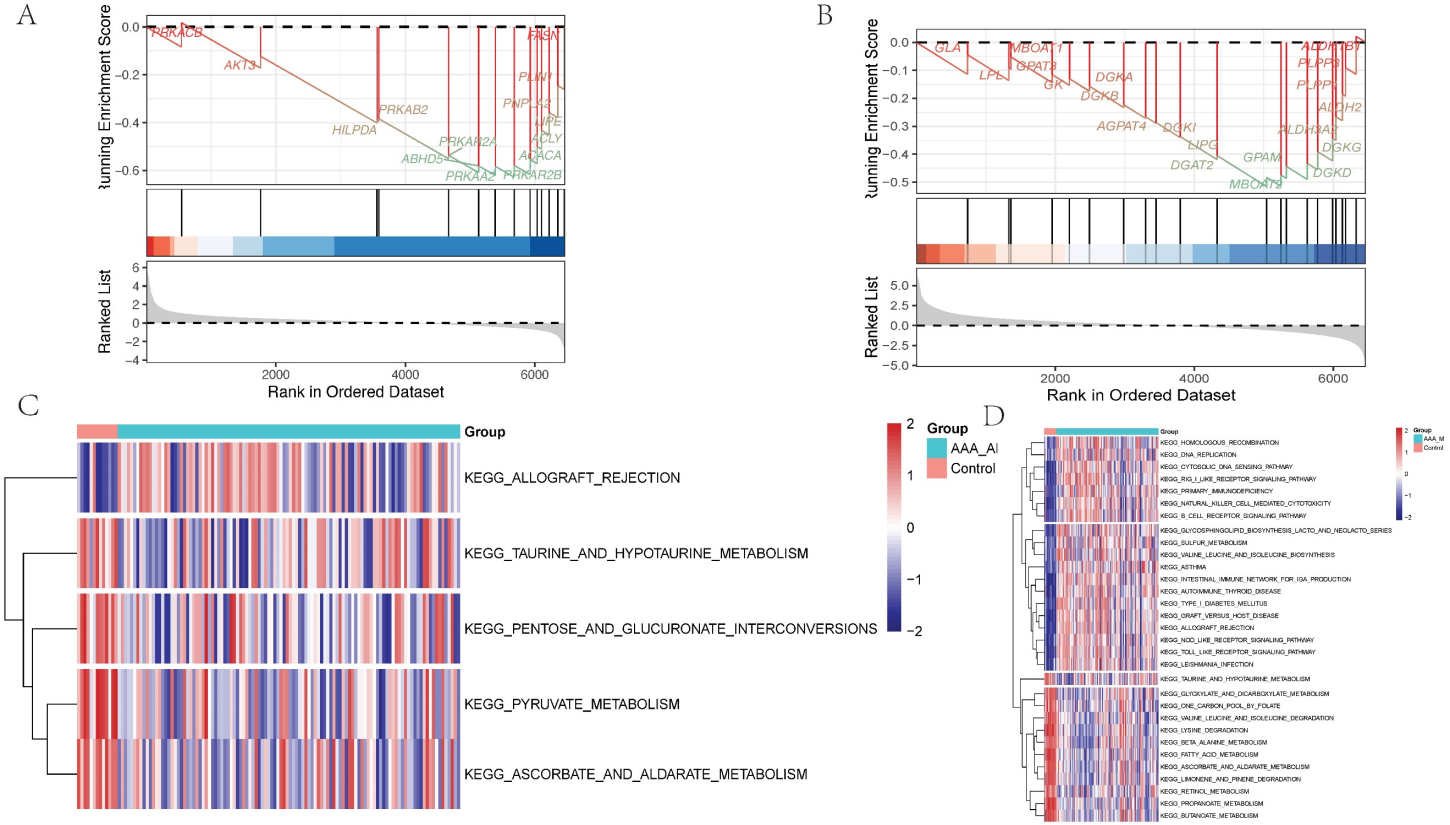
** Metabolism-related pathways are significantly enriched in the pathological progression of both the intima and adventitia of AAA.** 6A-B represent the GSEA distribution maps of taurine metabolism pathways enriched by differential genes in the vascular adventitia and intima, respectively; 6C-D display heatmaps of metabolic pathway changes obtained after quantifying the differential genes in the vascular adventitia and intima using GSVA.

GSEA of differential genes in the adventitia and intima highlighted core metabolic pathways. For the adventitia, GSEA suggested significant enrichment in taurine and hypotaurine metabolism (Counts = 14, Enrichment Score = -0.63, NES = -1.83, Adjusted p-value = 0.009) (Figure 6A). For the intima, GSEA indicated taurine and hypotaurine metabolism (Counts = 21, Enrichment Score = -0.52, NES = -1.50, Adjusted p-value = 0.037) (Figure 6B). Additionally, through Gene Set Variation Analysis (GSVA), quantified scoring of metabolic pathways revealed prominent differential pathways in the adventitia, including KEGG: PYRUVATE METABOLISM, KEGG: ASCORBATE AND ALDARATE METABOLISM, KEGG: PENTOSE AND GLUCURONATE INTERCONVERSIONS, KEGG: TAURINE AND HYPOTAURINE METABOLISM, and KEGG: ALLOGRAFT REJECTION. In contrast, the intima primarily included KEGG: HOMOLOGOUS RECOMBINATION, KEGG: BUTANOATE METABOLISM, KEGG: RETINOL METABOLISM, and KEGG: PROPANOATE METABOLISM (Figures 6C-D).

### Machine learning identification of core regulatory pathways and genes

This study evaluated the predictive performance of various machine learning classifiers in the classification of disease states using a dataset that included expressions of multiple genes related to specific metabolic pathways and immunological responses.

Of the media samples, the classifiers employed were XGB Classifier, Logistic Regression, RandomForest Classifier, AdaBoost Classifier, DecisionTree Classifier, GradientBoosting Classifier, Gaussian NB, SVC, and KNeighbors Classifier. Performance was assessed based on the area under the receiver operating characteristic curve (AUC), using 10-fold cross-validation. The RandomForest Classifier exhibited the highest predictive accuracy in the training dataset with an exemplary AUC of 1.000 (95% CI: 0.000), sensitivity of 1.000 (0.000), and specificity of 1.000 (0.000). The classifier also showed an excellent F1 score and Kappa coefficient, both reaching their maximum values of 1.000 (0.000) and 0.954 (0.002), respectively, suggesting potential overfitting to the training data. In contrast, the Gaussian Naive Bayes (GNB) Classifier demonstrated the best performance on the validation dataset, with an AUC of 0.973 (95% CI: 0.058). The cutoff value for GNB was 0.401 with a variance of 0.489, indicating a moderate discriminative threshold. The accuracy was 0.887 (0.082), sensitivity was 0.964 (0.073), and specificity was a perfect 1.000. The positive predictive value was notably high at 0.975 (0.038), though the negative predictive value was not calculable due to missing values. The F1 score and Kappa for GNB were 0.969 (0.054) and 0.502 (0.329), respectively, highlighting its reliability and moderate agreement beyond chance.

Of the adventitia samples, the RandomForest Classifier exhibited the highest predictive accuracy in the training dataset with an exemplary AUC of 1.000 (95% CI: 0.000), sensitivity of 1.000 (0.000), and specificity of 1.000 (0.000). The classifier also showed an excellent F1 score and Kappa coefficient, both reaching their maximum values of 1.000 (0.000) and 0.954 (0.002), respectively, suggesting potential overfitting to the training data. In contrast, the Gaussian Naive Bayes (GNB) Classifier demonstrated the best performance on the validation dataset, with an AUC of 0.973 (95% CI: 0.058). The cutoff value for GNB was 0.401 with a variance of 0.489, indicating a moderate discriminative threshold. The accuracy was 0.887 (0.082), sensitivity was 0.964 (0.073), and specificity was a perfect 1.000. The positive predictive value was notably high at 0.975 (0.038), though the negative predictive value was not calculable due to missing values. The F1 score and Kappa for GNB were 0.969 (0.054) and 0.502 (0.329), respectively, highlighting its reliability and moderate agreement beyond chance.

Through the analysis, key pathways and genes were identified as critical for predicting pathological changes in the aneurysmal tissues of the abdominal aorta. The core pathways include KEGG: ASCORBATE AND ALDARATE METABOLISM, KEGG: ALLOGRAFT REJECTION, and KEGG: TAURINE AND HYPOTAURINE METABOLISM. Additionally, the genes CCL4, CCL5, CD74, CXCL10, CXCL9, CXCR4, MS4A1, PIK3CG, and PTGS2 were pinpointed as central to the disease's progression. Models varied significantly in their parameter settings, critical for their performance optimization. Detailed parameters for each model were strategically chosen to balance complexity and predictive power. Statistical analysis included a robust comparison of ROC curves to evaluate comprehensive model performance. The potential overfitting observed in the RandomForest Classifier contrasts with the GNB's robust performance on the validation set, suggesting better generalizability and stability.

The study highlights the necessity of selecting models based on validation performance, rather than training performance alone, to ensure generalizability in clinical settings. The identification of specific metabolic pathways and key genes offers potential targets for therapeutic intervention and a deeper understanding of the molecular mechanisms underlying the disease. Future research could explore ensemble methods to enhance predictive accuracy and stability across different datasets.

### Partial correlation analysis and protein interaction analysis

**Figure 8:**
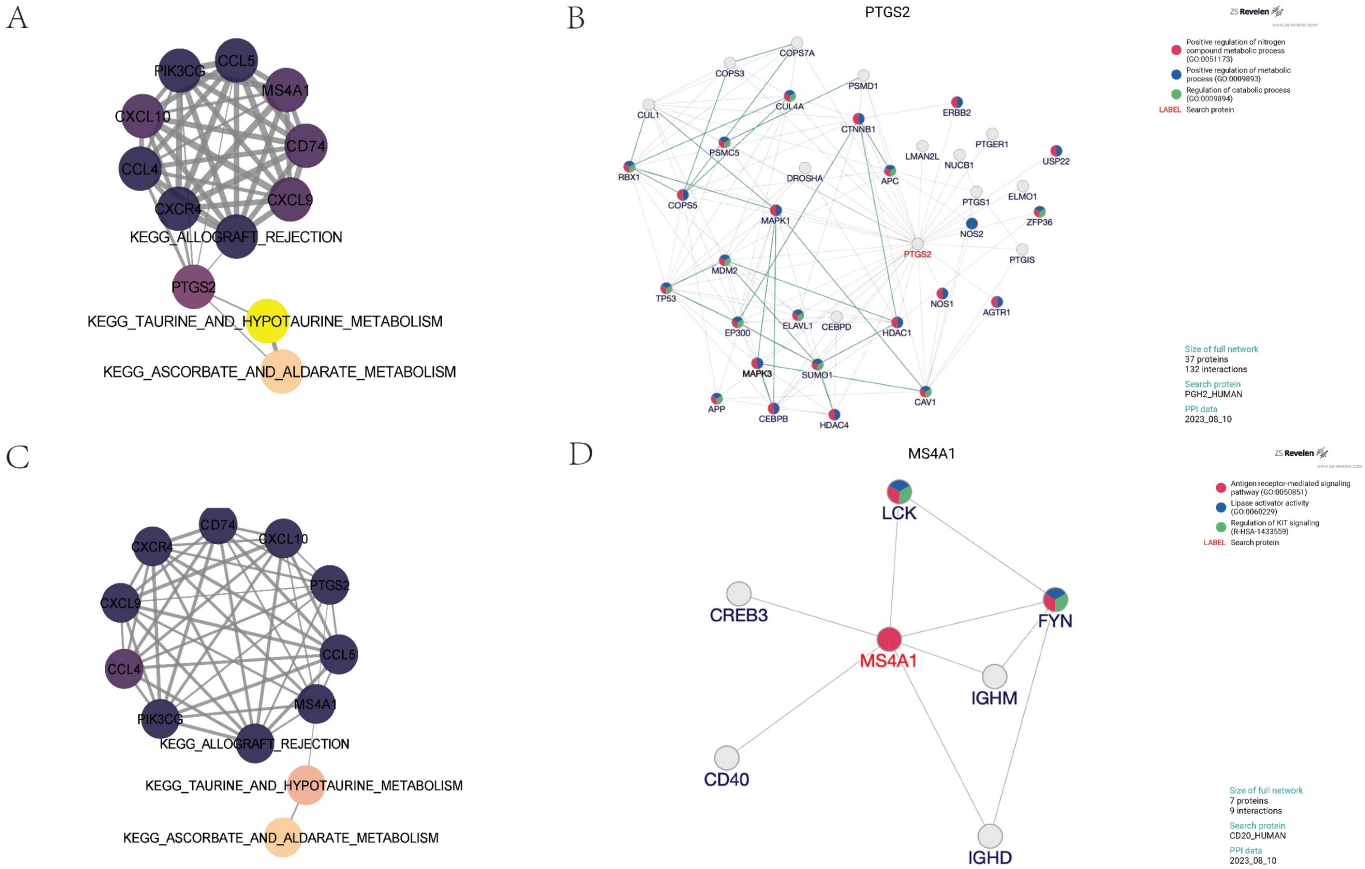
** Identification of core regulatory genes and construction of partial correlation interaction networks related to taurine metabolism pathways.** 8A shows the partial correlation interaction network related to taurine metabolism pathways associated with core gene expression in the vascular adventitia. 8B analyzes the interactions between the core regulatory gene PTGS2 and its upstream and downstream genes in the vascular adventitia. 8C displays the partial correlation interaction network related to taurine metabolism pathways associated with core gene expression in the vascular media. 8D analyzes the interactions between the core regulatory gene MS4A1 and its upstream and downstream genes in the vascular adventitia.

To further explore the core regulatory pathways quantitatively scored within the adventitia and intima tissues, we utilized partial correlation network analysis. We discovered that the PTGS2 gene in the adventitia acts as a principal regulator connecting the KEGG pathways for TAURINE AND HYPOTAURINE METABOLISM and ALLOGRAFT REJECTION with the expression of related inflammatory factors (Figure 8A). Molecular upstream and downstream regulatory analysis of PTGS2 revealed its close biological association with pathways such as Positive regulation of nitrogen compound metabolic process (GO:0051173), Positive regulation of metabolic process (GO:0009893), and Regulation of catabolic process (GO:0009894), suggesting its potential role as a regulator in TAURINE METABOLISM involved in adventitial pathology of abdominal aortic aneurysms (Figure 8B). Interestingly, in the analysis of the intima, MS4A1 was identified as the main regulator connecting KEGG_TAURINE_AND_HYPOTAURINE_METABOLISM, KEGG_ALLOGRAFT_REJECTION, and the expression of related inflammatory factors (Figure 8C). It is closely related to pathways such as Antigen receptor-mediated signaling pathway (GO:0050851), Lipase activator activity (GO:0060229), and Regulation of KIT signaling (R-HSA-1433559), suggesting its potential role as a regulator in TAURINE METABOLISM involved in intimal pathology of abdominal aortic aneurysms (Figure 8D).

## Discussion

Taurine constitutes nearly 50% of the free amino acid content in the heart, underscoring its vital role in cardiac biochemistry 32. It enhances myocardial contractility and modulates ionic balance, crucial for heart function. Taurine supplementation has been associated with improved cardiac output and left ventricular function in both animal models and human studies 33, 34. Notably, taurine's approval in Japan for the treatment of heart failure highlights its clinical relevance and therapeutic potential 32, 33.

Taurine influences cardiac function through several mechanisms: (1) Ion Handling: It regulates intracellular Ca^2+^ and Na^+^ levels, essential for proper cardiac rhythm and contractility. (2) Energy Metabolism: Taurine supports myocardial energetics, enhancing ATP production and energy utilization within cardiac cells. (3) Signaling Pathways: It interacts with multiple cellular signaling pathways, including those related to glucose transport and anti-inflammatory responses, thereby protecting the heart under stress conditions. (4) Hormonal Regulation: Taurine promotes natriuresis and diuresis, affecting fluid balance and blood pressure through its actions on renal function and hormone release 32-35.

Vascular endothelial health is crucial for maintaining vascular tone and integrity. Taurine enhances endothelial function by promoting nitric oxide (NO) production, which facilitates vascular relaxation and reduces arterial stiffness 36, 37. This action is particularly beneficial in preventing and managing vascular pathologies such as atherosclerosis. Taurine's ability to scavenge reactive oxygen species protects endothelial cells from oxidative stress, a key factor in the pathogenesis of vascular diseases. Clinical studies and meta-analyses have demonstrated that taurine supplementation can effectively reduce blood pressure, which is a significant risk factor for vascular disease. By enhancing endothelial cell function and reducing inflammatory responses, taurine helps maintain the endothelium's integrity and responsiveness, crucial for vascular health. While the existing research underscores taurine's potential in cardiovascular therapy, particularly for vascular pathologies, more extensive clinical trials are required to establish optimal dosing strategies, long-term effects, and mechanisms of action 37-39.

PTGS2, commonly known as cyclooxygenase-2 (COX-2), is increasingly recognized for its pivotal role in the pathogenesis of vascular diseases, particularly atherosclerosis 40-42. As an inducible enzyme predominantly expressed during inflammatory responses, PTGS2 catalyzes the conversion of arachidonic acid to prostaglandin H2 (PGH2), a precursor to various prostanoids involved in inflammatory and thrombotic processes. The expression of PTGS2 in vascular endothelial cells and macrophages is stimulated by inflammatory cytokines, growth factors, and mechanical stress, which are prevalent in atherosclerotic lesions 40-42. PTGS2-derived prostanoids, such as prostaglandin E2 (PGE2) and thromboxane A2 (TXA2), contribute to vascular inflammation by enhancing leukocyte recruitment, vascular permeability, and platelet aggregation. Furthermore, PTGS2 is implicated in the modulation of endothelial function and vascular smooth muscle cell migration and proliferation, key events in the development of atherosclerotic plaques. Clinical and experimental studies have highlighted the dual role of PTGS2 in promoting initial inflammatory responses and in the resolution phase of inflammation, suggesting a complex involvement in the progression and stabilization of atherosclerotic lesions 41, 43. Given its central role in mediating inflammatory responses within vascular tissues, PTGS2 represents a potential therapeutic target for modulating disease progression in atherosclerosis and possibly other related vascular disorders. Consequently, selective PTGS2 inhibitors and nonsteroidal anti-inflammatory drugs (NSAIDs) targeting COX pathways are under investigation for their capacity to mitigate inflammatory processes in the vasculature without adverse cardiovascular effects 40, 42.

MS4A1, commonly known as CD20, plays a pivotal role in the pathogenesis of Kawasaki Disease (KD), a principal cause of acquired pediatric heart disease characterized by vasculitis primarily affecting the coronary arteries. This condition can lead to the formation of coronary artery aneurysms, significantly elevating the risk of myocardial infarction and ischemic heart diseases in children 44, 45. MS4A1 is a key marker of B-cell activation and differentiation, essential for the immune system's response through antibody production. Its elevated expression in KD suggests a robust immune activation contributing to the disease's vascular pathology. Recent immunohistochemical studies have shown significant upregulation of MS4A1 alongside other immune markers such as AIF1, IL-18, and TLR-7 in the coronary tissues of KD fatalities 44, 45. This suggests a dynamic interplay among various immune cells, including macrophages, dendritic cells, and B-cells within the inflamed vascular tissues. Notably, the co-localization of MS4A1 with macrophage and dendritic cell markers highlights complex cellular interactions that exacerbate arterial inflammation, leading to severe vascular damage 44, 46. The presence of MS4A1-positive B-cells within inflamed tissues indicates their dual role in KD pathogenesis: directly, through proliferation and aberrant activation; and indirectly, by facilitating other immune responses via antigen presentation and cytokine production. Furthermore, the correlation between MS4A1 expression levels and disease severity underscores its potential as both a biomarker of disease activity and a prognostic indicator, providing insights into the severity of coronary artery aneurysms. Therapeutically, the significant role of MS4A1 in KD raises the possibility of developing targeted interventions to modulate its activity. Analogous to the use of anti-CD20 monoclonal antibodies like Rituximab in other autoimmune conditions, targeting MS4A1 could mitigate the excessive immune response characteristic of KD, thereby ameliorating coronary lesions and improving cardiovascular outcomes. In summary, MS4A1/CD20 is integral to the complex immune landscape of KD. Its study not only enriches our understanding of KD's immunopathology but also facilitates the exploration of targeted therapies 45, 46. Such advances are crucial for enhancing the treatment paradigms of this severe pediatric condition, emphasizing the need for continued research into immunological markers in vascular inflammatory diseases, especially in pediatric cohorts where early detection and therapeutic intervention are critical.

## Conclusion

This study delineates the roles of MS4A1 and PTGS2 in taurine metabolic reprogramming and their implications in the pathogenesis of AAA. In addition, this study not only advances our understanding of the complex pathophysiology associated with AAA but also lays the groundwork for future research into targeted treatments. By bridging advanced bioinformatics tools with deep molecular insights, we pave the way for innovative strategies in the prevention and management of AAA, potentially transforming patient outcomes in vascular health.

## Funding

This study is supported by the National Natural Science Foundation of China (No. 82241209).

## Figures and Tables

**Figure 4:**
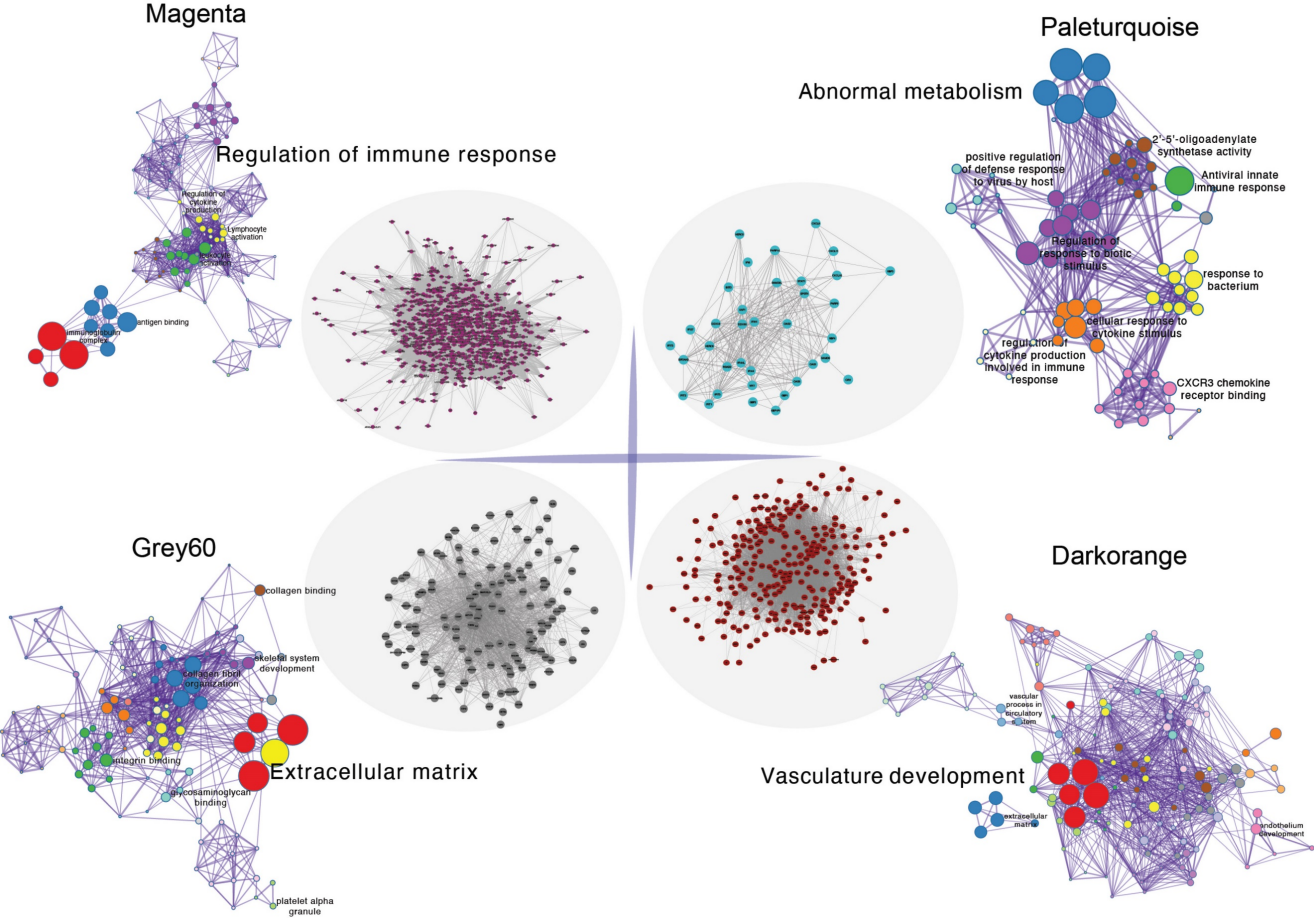
The protein-protein interactions within the core gene module and enrichment of key pathways.

**Figure 7:**
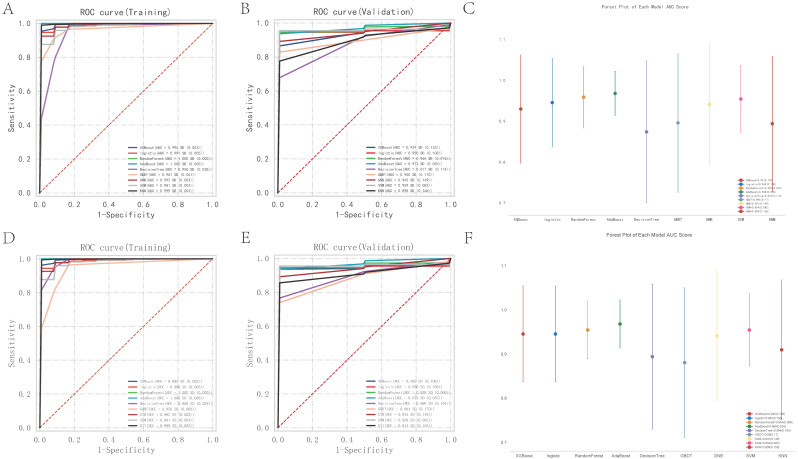
** Nine machine learning classification algorithms for the identification of core regulatory genes and pathways in the adventitia and intima of abdominal aortic aneurysms.** 7A-B represent the ROC indices for core genes and pathways in the training and validation sets for the vascular adventitia, respectively. 7C displays a forest plot of model characteristics for the nine machine learning algorithms used for the vascular adventitia. 7D-E show the ROC indices for core genes and pathways in the training and validation sets for the vascular media, respectively, while 7F presents a forest plot of model characteristics for the nine machine learning algorithms used for the vascular media.
